# Chemical and Genetic Discrimination of Cistanches Herba Based on UPLC-QTOF/MS and DNA Barcoding

**DOI:** 10.1371/journal.pone.0098061

**Published:** 2014-05-22

**Authors:** Sihao Zheng, Xue Jiang, Labin Wu, Zenghui Wang, Linfang Huang

**Affiliations:** Institute of Medicinal Plant Development, Chinese Academy of Medical Sciences, Peking Union Medical College, Beijing, People's Republic of China; University of Milano Bicocca, Italy

## Abstract

Cistanches Herba (Rou Cong Rong), known as “Ginseng of the desert”, has a striking curative effect on strength and nourishment, especially in kidney reinforcement to strengthen yang. However, the two plant origins of Cistanches Herba, *Cistanche deserticola* and *Cistanche tubulosa*, vary in terms of pharmacological action and chemical components. To discriminate the plant origin of Cistanches Herba, a combined method system of chemical and genetic –UPLC-QTOF/MS technology and DNA barcoding–were firstly employed in this study. The results indicated that three potential marker compounds (isomer of campneoside II, cistanoside C, and cistanoside A) were obtained to discriminate the two origins by PCA and OPLS-DA analyses. DNA barcoding enabled to differentiate two origins accurately. NJ tree showed that two origins clustered into two clades. Our findings demonstrate that the two origins of Cistanches Herba possess different chemical compositions and genetic variation. This is the first reported evaluation of two origins of Cistanches Herba, and the finding will facilitate quality control and its clinical application.

## Introduction

Cistanches Herba (Rou Cong Rong), known as “Ginseng of the desert”, originates from dried succulent stems of *Cistanche deserticola* Y.C. Ma and *Cistanche tubulosa* (Schrenk) Wig according to the Chinese Pharmacopoeia (2010 edition), and is popular for its tonifying the kidney-yin, benefiting life essence and relaxing bowel. Currently, Cistanches Herba is mainly distributed in arid and warm deserts in northwest China, particularly in Xinjiang and Inner Mongolia provinces. However, the two origins of Cistanches Herba differ in terms of their pharmacological activity and chemical components. Tu et al. investigated the decoction of three *Cistanche* species (*C. deserticola*, *C. tubulosa*, *Cistanche salsa*) and found that *C. tubulosa* showed the lowest effect in the Yang-deficiency mouse model [1]. Zhang et al. compared pharmacological activity between *C. deserticola*, *C. tubulosa* and *C. salsa*, and found that these species had medicinal functions such as anti-fatigue and hypoxia tolerance, but not on the same extent [2]. Previous research reported the chemical component, and indicated the difference of chemical component and content for plant origins of Cistanches Herba [3]. As for the clinical application and market circulation,as a tonic,*C. tubulosa* has been traditionally used as a blood circulation-promoting agent and in the treatment of impotence, sterility, lumbago, body weakness in Japan [4]–[8].

Consequently, it is of great significance to discriminate two origins of Cistanches Herba for the quality control and clinical application. However, there is no research focus on discrimination of two origins of Cistanches Herba. Many researched methods, including microscopy, ultraviolet and infrared detection, inter-simple sequence repeats method have been used to identify the genus of Cistanches, but not only for two origins specially [9]–[20]. Here, we conjunctively utilized chemical and molecular techniques to distinguish two origins of Cistanches Herba, UPLC-QTOF/MS (ultra-performance liquid chromatography coupled with quadrupole time-of-flight mass spectrometry) and DNA barcoding. UPLC-QTOF/MS provides information more rapidly and efficiently compared with other techniques. The high selectivity and sensitivity of UPLC-QTOF/MS have resulted in its application for both quantitative and qualitative analyses, as well as in metabolite analysis and identification of complex compounds in Traditional Chinese Medicine [21]–[22]. Principal component analysis (PCA) and orthogonal projection to latent structure-discriminant analysis (OPLSDA) are also developed to identify potential marker compounds. DNA barcoding, an easier and more universal molecular marker technology, uses a DNA fragment to identify species or genera. It is objective, more accurate, and easier to perform than traditional identification methods and other molecular marker technologies. Moreover, DNA barcoding has successfully been applied to identify animal and plant, including medicinal plants [23]–[26].

The purpose of this research is to establish a scientific method system, combined UPLC-QTOF/MS and DNA barcoding, for discrimination of two plant origins of Cistanches Herba.

## Materials and Methods

### Ethics statement

We confirm that the field studies did not involve endangered or protected species. GPS coordinates have included in the sample information, please see table 1."

**Table 1:** Samples of *Cistanche deserticola* and *Cistanche tubulosa*.

No.	Family	Species	Sources	GPS coordinates	GenBank accession number
R11-R13	Orobanchaceae	*C. deserticola*	WLMQ Xinjiang	E:87.69° W:43.75°	KF289954–KF289956
R21-R23	Orobanchaceae	*C. deserticola*	GJH Xinjiang	E:83.37° W:44.81°	KF289957,KF289958(R22)
R51-R56	Orobanchaceae	*C. deserticola*	HBKSEMG Xinjiang	E:85.55° W:46.69°	KF574740–KF574745
R61-R66	Orobanchaceae	*C. deserticola*	KLMY Xinjiang	E:84.84° W:45.59°	KF574746–KF574749(R61,R62)
R71-R82	Orobanchaceae	*C. deserticola*	BDJLSM Inner-Mongolia	E:102.62°W:39.72°	KF574750–KF574760(R79)
R31-R33	Orobanchaceae	*C. deserticola*	ALSZQ Inner-Mongolia	E:105.63°W:38.83°	KF289959–KF289961
R41-R43	Orobanchaceae	*C. deserticola*	BDJLSM Inner-Mongolia	E:102.16°W:39.66°	KF289965–KF289964
G11-G13	Orobanchaceae	*C. tubulosa*	DSX Xinjiang	E:88.82° W:43.87°	KF289945–KF289947
G21-G22	Orobanchaceae	*C. tubulosa*	CL Xinjiang	E:80.83° W:36.96°	KF289948,KF289949
G31-G33	Orobanchaceae	*C. tubulosa*	MF Xinjiang	E:82.69° W:37.08°	KF289950–KF289952
G41-G43	Orobanchaceae	*C. tubulosa*	HT Xinjiang	E:79.80° W:37.03°	KF289953(R41,R42)

WLMQ meant Wu Lu Mu Qi city; GJH meant Gan Jia Hu; HBKSEMG meant Hoboksar Mongol Autonomous County; KLMY meant Ke La Ma Yi city; BDJLSM meant Badain Jaran Desert; ALSZQ meant Alxa Left Banner; DSX meant Dong San county; CL meant Ce Le county; MF meant Min Feng county; HT meant He Tian county.

### Plant materials and reagents

Succulent stems of Cistanches Herba were collected from wild desert region in Inner Mongolia, Qinghai Provinces, Xinjiang Uygur Autonomous Region, People's Republic of China (Table 1) in May 2012. The samples of the research were all collected in wild desert region, not in private land, where no specific permissions were required. The botanical identities of the stems were confirmed by Dr. Linfang Huang. Voucher specimens were deposited at The Institute of Medicinal Plant Development. High-performance liquid chromatography (HPLC)-grade acetonitrile (Merck KGaA, Darmstadt, Germany) and formic acid (Tedia, USA) were utilized for UPLC analysis. Deionized water was purified using a Milli-Q system (Millipore, Bedford, MA, USA). All other chemicals were of analytical grade.

### Sample preparation

Cistanches Herba samples (1.0 g, 65-mesh) were transferred into a 50-mL conical flask, and 50 mL of 70% methanol was added. After soaking for 30 min, ultrasonication (35 kHz) was performed at room temperature for 30 min. After centrifugation at 10,000 rev/min for 10 min, the supernatant was stored at 4°C and filtered through a 0.22-μm membrane before injection into the UPLC-QTOF/MS system for analysis.

### UPLC-QTOF/MS

For UPLC analysis, the following systems/parameters were used: Waters Acquity system (Waters) equipped with a binary solvent delivery pump, auto-sampler and PDA detector connected to a Waters Empower 2 data station; ultrasonication (250 W, 50 kHz, Kunshan Ultrasonic Instrument Co., Zhejiang, China); and an electronic analytical balance model AB135-2 (Mettler-Toledo., Greifensee, Zurich, Switzerland). A Waters Acquity UPLC BEH C_18_ column (1.7 µm, 2.1×100 mm, Waters) and a Waters C_18_ guard column (same material, waters) were used and maintained at 30°C. The mobile phase was 0.1% formic acid aqueous solution (A) and acetonitrile (B) with a gradient program as follows: 0–3 min, 10–22% B; 3–4 min, 22–23% B; 4–6 min, 23–35% B; 6–8 min, 35–37% B; 8–11 min, 37–42% B; 11–12 min, 42–48% B; 12–15 min, 48%–50% B at a flow rate of 0.3 mL/min. The injection volume was 5 µL.

The UPLC/MS analysis was performed on a QTOF Synapt G2 HDMS system (Waters, Manchester, UK) equipped with an electrospray ionization (ESI) source operated in the negative-ion mode. N_2_ was used as the desolvation gas. The desolvation temperature was set at 450°C at a flow rate of 800 L/h, and the source temperature was set at 120°C. The capillary and cone voltages were set to 2500 and 40 V, respectively. Data were collected between 50–1200 Da with a 0.1-s scan time and a 0.01-s interscan delay over a 15-min analysis time. Argon was used as the collision gas at a pressure of 7.06661023 Pa. All MS data were collected using the LockSpray system to ensure mass accuracy and reproducibility. The [M-H]^-^ ion of leucine-enkephalin at m/z 554.2615 was used as the lock mass in negative ESI mode.

### Data analysis

UPLC-QTOF/MS data for Cistanches Herba samples were analyzed to identify potential discriminant variables. Peak finding, alignment and filtering of ES raw data were carried out using the Marker Lynx applications manager, version 4.1 (Waters, Manchester, UK). The parameters used were as follows: retention time (t_R_) of 0–15 min, mass of 50–1200 Da, retention time tolerance of 0.02 min, and mass tolerance of 0.02 Da. Three replicate samples collected from each geographic location were used (n = 3). A total of 6, 339 variables were used to create the model.

### DNA barcoding: DNA extraction, PCR amplification and sequencing

Samples taken from dried fleshy stems of *C. deserticola* and *C. tubulosa* (30 mg) were rubbed for 2 minutes at a frequency of 30 r/s. DNA was extracted according to the manufacturer's instructions (Tiangen). Specifically, the protocol was modified such that chloroform was replaced with a mixture of chloroform: isoamyl alcohol (24∶1 in the same volume), and buffer solution GP2 with isopropanol (same volume). The rubbed powder was put into 1.5 ml eppendorf tubes, added 700 µL 65°C preheated GP1 and 1 µL β-mercaptoethanol to mix using vortex for 10–20 s, and incubated for 60 minutes at 65°C; Adding 700 µL mixture of chloroform: isoamyl alcohol (24∶1), centrifuge for 5 minutes at 12000 rpm(∼13400×g); Pipette supernatant to a new tube, adding 700 µL isopropanol, blending for 15–20 minutes; Piping all the mixture into spin column CB3 and centrifuge for 40 s at 12000 rpm; Discarding the filtrate and adding 500 µL GD(adding quantitative anhydrous ethanol before use), centrifuge at 12000 rpm for 40 s; discarding the filtrate and adding 700 µL PW(adding quantitative anhydrous ethanol before use) to wash the membrane, centrifuge for 40 s at 12000 rpm; Discarding the filtrate and adding 500 µL PW, centrifuge for 40 s at 12000 rpm; Discarding the filtrate and centrifuge for 2 minutes at 12000 rpm to remove residual wash buffer PW; Transferring the spin column CB3 into a clean 1.5 ml eppendorf tube, and drying at room temperature for 3–5 minutes; Centrifuge for 2 minutes at 12000 rpm to obtain the total DNA. Primers for polymerase chain reaction (PCR) were based on sequences reported previously [4], [5]. PCR reaction mixtures contained 2-μL DNA template, 8.5-μL ddH_2_O, 12.5-μL 2× Taq PCR Master Mix (Beijing TransGen Biotech Co., China), 1/1-μL forward/reverse (F/R) primers (2.5 µM), in a final volume of 25 µL. PCR amplification was conducted as described by Kress et al. [4]. The primer of PCR reaction were fwd PA: GTTATGCATGAACGTAATGCTC (5′-3′) and rev TH: CGCGCATGGTGGATTCACAATCC (5′-3′). PCR products were separated and detected by 1% agarose gel electrophoresis. PCR products were purified following the manufacturer's protocol and directly subjected to sequencing.

### Sequence alignment and analysis

ITS and ITS2 sequences were collected from the GenBank database. Sequences from sequencing of the samples were submitted to GenBank database (Accession numbers were listed in table 1), assembled with CodonCode Aligner 3.7.1 (CodonCode Co., USA) and aligned using ClustalW. Kimura 2-Parameter (K2P) distances, GC content of base and Neighbor-joining (N-J) trees were calculated and constructed using the MEGA 5.05 with the Bootstrap method (1000 resampling) and K2P model [27]. Barcoding gap (spacer region that was formed between intra- and inter-specific genetic variations) and identification efficiency (the ability of identification for comparing different barcodes) were drawn and calculated based on the method reported by Meyer and Paulay [28].

## Results

### Tentative peak assignment by UPLC-QTOF/MS

**Figure 1:**
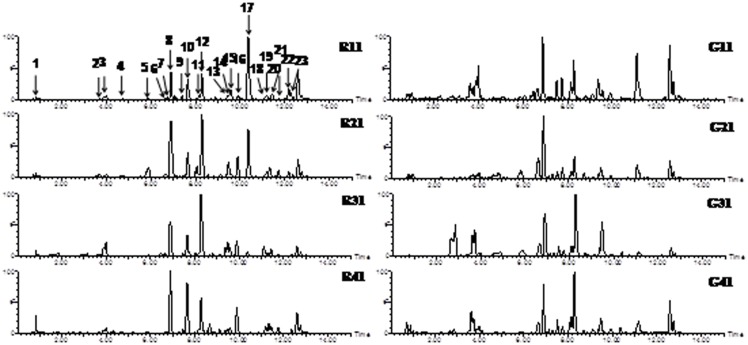
The representative chromatograms of *C. deserticola* and *C. tubulosa.* The left side were the chromatograms of *C. deserticola* collected from different locations; the right side were the chromatograms of *C. tubulosa* collected from different locations. This figure shows the differences between these two origins in chemical profiles.

Representative chromatograms of *C. deserticola* and *C. tubulosa* from different producing areas are shown in Figure 1. The fingerprint chromatogram indicated similarities among Cistanches Herba samples. A total of 23 qualified mass peaks were detected and 16 peaks were identified by matching the retention times and mass spectra with those reported previously (Table 2) [29]–[35]. Peaks 2, 3, 5, 6, 7, 8, 9, 11, 12, 15, 16, 17, 20, 21, 22, and 23 were tentatively identified as cistanoside F, mussaenoside acide, cistanbuloside C1/C2, campneoside II, isomer of campneoside II, echinacoside, cistanoside A, acteoside, isoacteoside, syringalide A-3′-α-L-rhamnopyranoside, cistanoside C, 2′-acetylacteosid, osmanthuside B, cistanoside D, tubuloside B, and cistancinenside A, respectively. Chemical constituents were determined to be primarily phenylethanoid glycosides (PhGs), while one compound, mussaenoside acide, was an iridoid polysaccharide. PhGs are the main active compounds in terms of treatment of kidney deficiency, and antioxidant and neuroprotective effects [36].

**Table 2:** Tentatively identified compounds from *C. deserticola* and *C. tubulosa.*

Peak no.	t_R_ (min)	Name	Formula	[M-H]^−^ m/z			Fragments m/z	Reference
				Mean measured mass(Da)	Theoretical exact mass(Da)	ppm		
1	0.84	unknown	C_12_H_22_O_11_	341.1078	341.1084	−1.8	195.0512, 179.0585	
2	3.69	cistanoside F	C_21_H_28_O_13_	487.1454	487.1452	0.4	179.0355	[13]
3	4.00	mussaenoside acide	C_16_H_24_O_10_	375.1289	375.1291	−0.5	235.0561, 213.0758, 191.0689,169.0860	[14]
4	4.87	unknown	C_54_H_28_O	691.2054	691.2062	−1.2	307.0252, 161.0243	
5	5.89	cistanbulosideC1/C2	C_35_H_46_O_21_	801.2449	801.2453	−0.5	621.2043, 201.0146, 179.0353, 161.0230	[15]
6	6.58	campneoside II	C_47_H_28_O_3_	639.1947	639.1960	−2.0	621.2183,179.0359,161.0235	[16]
7	6.66	Isomer of campneoside II	C_47_H_28_O_3_	639.1947	639.1960	−2.0	621.2183,179.0359,161.0235	[16]
8	6.89	echinacoside	C_35_H_46_O_20_	785.2517	785.2504	1.7	623.2201,477.1612,461.1643,161.0244,135.0445	[16]–[18]
9	7.64	cistanoside A	C_36_H_48_O_20_	799.2673	799.2661	1.5	637.2346,623.2080,491.1746,161.0241	[16]–[18]
10	7.51	unknown	C_22_H_38_O_12_	493.2274	493.2285	−2.2	–	
11	8.28	acteoside	C_29_H_36_O_15_	623.1989	623.1976	2.1	421.1646,319.0787,161.0237	[16]–[18]
12	9.48	isoacteoside	C_29_H_36_O_15_	623.1989	623.1976	2.1	421.1646,319.0787,161.0237	[16]–[18]
13	8.78	unknown	C_16_H_26_O_8_	345.1552	345.1549	0.9	205.0836,179.0338,161.0226	
14	9.36	unknown	C_16_H_26_O_8_	345.1548	345.1549	−0.3	247.0938, 205.0836, 165.0902161.0236	
15	9.57	syringalide A-3′-α-L-rhamnopyranoside	C_29_H_36_O_14_	607.2023	607.2027	−0.7	445.1716,299.1123,201.0165, 161.0235	[16]
16	9.89	cistanoside C	C_30_H_38_O_15_	637.2137	637.2132	0.8	475.1777, 461.1655,315.1072,179.0344,161.0245	[16]
17	10.35	2′-acetylacteosid	C_31_H_38_O_16_	665.2086	665.2082	0.6	645.1805,623.2011,503.1779,461.1668,315.1097179.0339,161.0242	[16]–[18]
18	11.09	unknown	C_16_H_28_O_8_	347.1076	347.1706	0	369.1531, 347.1715, 207.1012, 161.0245	
19	11.31	unknown	C_30_H_38_O_15_	637.2135	637.2132	0.5	637.2114, 591.2069, 487.3010, 451.3267	
20	11.43	osmanthuside B	C_29_H_36_O_13_	591.2076	591.2078	−0.3	467.1423,45.1707,161.0224,145.0284	[16]
21	11.74	cistanoside D	C_31_H_40_O_15_	651.2287	651.2289	−1.7	677.4901, 527.1442, 351.1073, 175.0393	[19]
22	12.17	tubuloside B	C_31_H_38_O_16_	665.2086	665.2082	0.6	461.1709, 161.0244	[16]
23	12.38	Cistancinenside A	C_32_H_40_O_16_	679.2253	679.2238	2.2	677.4927, 659.1908, 179.0355, 161.,0236	[15]

### *PCA of* C. deserticola *and* C. tubulosa

**Figure 2:**
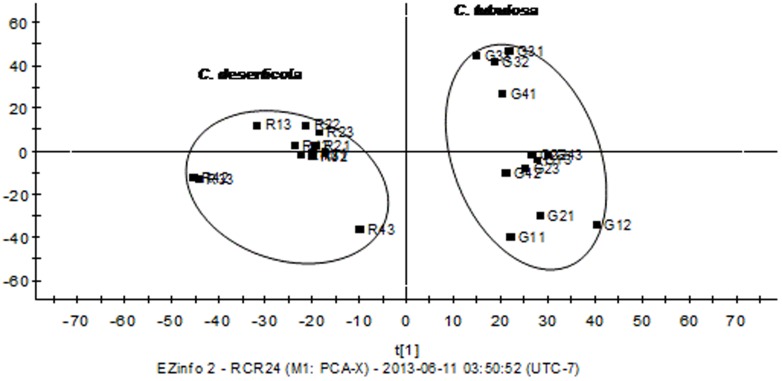
PCA of *C. deserticola* and *C. tubulosa*. These samples were clustered into two groups according to their species origin, which indicated that the chemical composition between *C. deserticola* and *C. tubulosa* were significantly different.

PCA was employed to distinguish samples of different plant species. PCA is an unsupervised multivariate data analysis method that aims to visualize the similarities and/or differences within multivariate data of secondary metabolite composition [37]. The two-component PCA model cumulatively accounted for 46.04% of the variation (PC1, 36.43%; PC2, 9.61%). Figure 2 shows that 24 samples were clustered into two groups in the PCA scores plotted according to species origin, indicating that the chemical composition of *C. deserticola* and *C. tubulosa* differed significantly.

### OPLS-DA and marker identification

**Figure 3:**
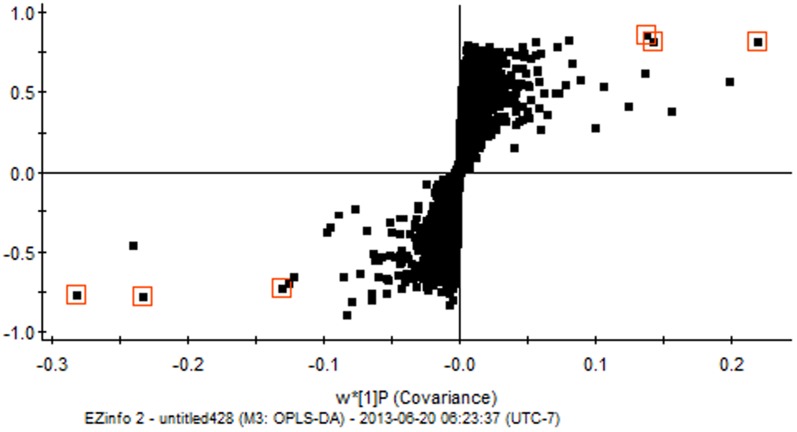
OPLS-DA (S-plot) of *C. deserticola and C. tubulosa*. One plot represents one t_R_–m/z ion pair. These square plots were the chemical marker ions found to distinguish two origins. The square plots in third quadrant were the chemical marker ions with higher contribution and confidence in *C. deserticola*, and the square plots in first quadrant were the chemical marker ions with higher contribution and confidence in *C. tubulosa*.

To identify potential chemical markers for discrimination of the two species, the S-plot of OPLS-DA was generated (Fig. 3). In the S-plot, each point represents one t_R_–m/z ion pair. The X and Y axes represent the contribution and confidence of the ion, respectively; the farther the distance the ion *t*_R_–*m*/*z* pair points from zero, the larger the contribution/confidence of this ion is to the difference between the two groups. Thus, the t_R_–m/z ion pointing to the two ends of the ‘S’ represent the characteristic markers with the highest confidence in each group.

**Figure 4:**
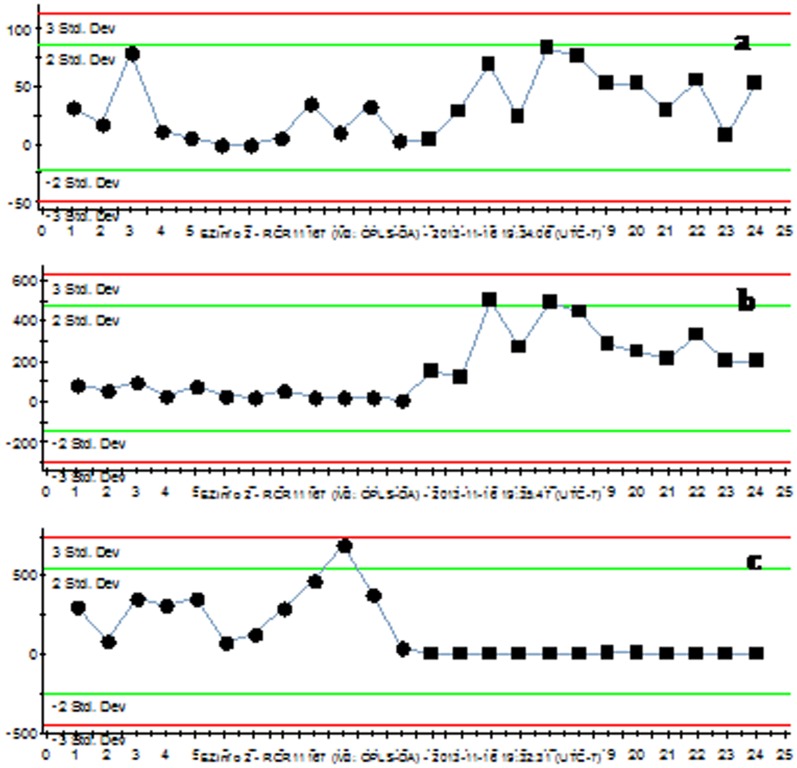
Ion intensities of markers a, b and c. The ion intensities of marker ions **a** and **b** in *C. deserticola* was higher than that in *C. Tubulosa*, and the marker ion **c** could be detected in *C. tubulosa*, but could not be detected in *C. deserticola*.

The OPLS-DA results showed that UPLC-QTOF/MS could be used to distinguish *C. deserticola* from *C. tubulosa* (Fig. 3). A total of six credible and significant markers were determined to facilitate discrimination of these groups (Table 3). The identities of three potential markers were tentatively assigned. The components correlated with these three ions were tentatively identified as isomers of campneoside II, cistanoside C and cistanoside A. The marker compounds **a**, **b** and **c** could be used to distinguish the two plant species, as the ion intensities of **a** and **b** in *C. deserticola* was higher than in *C. tubulosa* (Fig. 4A, 4B), and marker **c** could be detected in *C. tubulosa*, but not in *C. deserticola* (Fig. 4C).

**Table 3:** Marer t_R_–m/z ion pairs in the S-plot.

Species	Marker ions
*C. deserticola*	7.64–799.2667**a**; 9.88–637.2135**b**; 0.84–341.1081
*C. tubulosa*	6.66–639.1929**c**; 8.15–493.2284; 4.27–405.0499

### DNA barcoding: sequence information and identification efficiency

Sequence information was shown in Table 4. The average genetic distance of *psb*A-*trn*H (0.1732) was larger than other two regions (0.0740, 0.1197) significantly. The average GC content of *psb*A-*trn*H (20.64%) was smaller than other two regions (55.00%, 55.00%). Though the success rate of ITS and ITS2 was not obtained in this study, the *psb*A-*trn*H region performed well in PCR amplification and sequencing (100%, 87.23%). Identification efficiency was achieved by BLAST1 analysis and the nearest-distance method, and mainly reflected the success rate of the barcodes. The *psb*A-*trn*H region was clearly higher than the other two barcodes in identification efficiency based on two methods. The shortage of sequences is most likely the reason that ITS region exhibited 100% identification efficiency based on BLAST1 method, and 0 based on the nearest-distance method.

**Table 4:** Identification efficiency of three loci using different methods for species identification.

Markers		ITS	ITS2	*psb*A-*trn*H
Number of sequences		4	18	37
Length range/bp		617–622	235–236	358–558
Average GC content		55.00%	55.00%	20.64%
Efficiency of PCR amplification/%		/	/	100
Success rate of sequencing/%		/	/	87.23
Genetic distance	Min	0.0016	0.0000	0.0000
	Max	0.1280	0.2379	0.5279
	Average	0.0740	0.1197	0.1732
Identification efficiency/%	BlAST1	5.56	0	29.73
	Nearest Distance	100	5.56	100

### Analysis of genetic divergence using six parameters

Six parameters were used to analyze intra-specific variation and inter-specific divergence using three barcodes (Table 5). The significant difference between inter- and intra-specific variations was indicative of the utility of the DNA barcodes. Here, the minimum interspecific distance of three barcodes was all higher than the maximum intraspecific distance. Moreover, *psb*A-*trn*H region had larger maximum intraspecific distance and average interspecific distance than the other two barcodes, indicated that *psb*A-*trn*H region performed well in discrimination of two origins of Cistanches Herba.

**Table 5:** Analysis of interbygenus-specific divergence and intra-specific variation of three barcodes.

Markers	ITS	ITS2	psbA-trnH
Theta (avg_intra_avg)	0.0225	0.0000	0.0091±0.0019
coalescent depth (avg_intra_max)	0.0338	0.0000	0.0539±0.0013
All intraspecific distance (avg_between_intra-species)	0.0225±0.0181	0.0000	0.0102±0.0100
Theta prime (avg_interbyG_avg)	0.1258±0.0181	0.2381	0.4090±0.0100
minimum interspecific distance (avg_interbyG_min)	0.1235±0.0181	0.2381	0.2345±0.0100
all inter-specific distance (avg_between_interbyG)	0.1258±0.0025	0.0000	0.4090±0.0476

### Analysis of barcoding gap to identify C. deserticola and C. tubulosa

**Figure 5:**
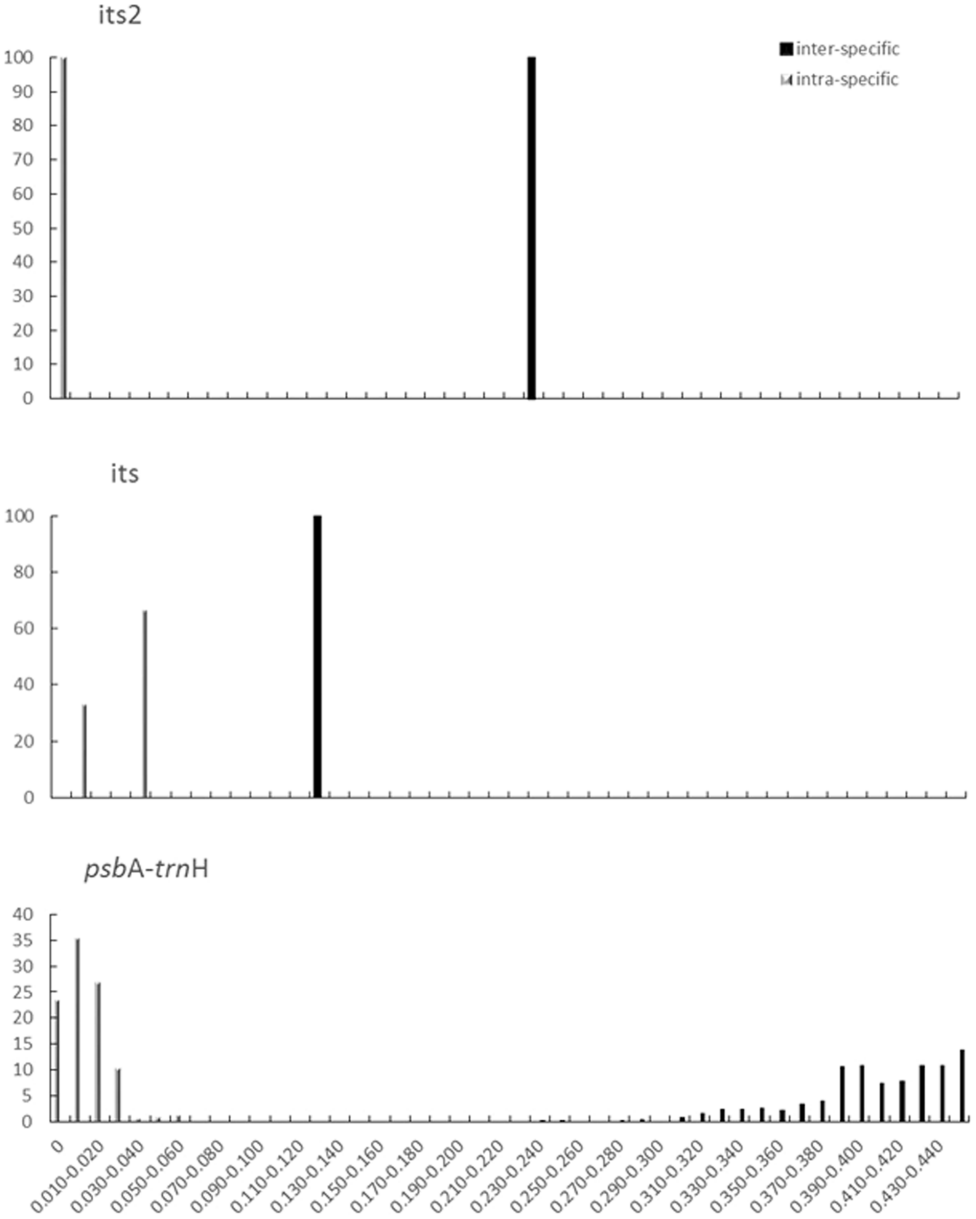
Relative distribution of inter-specific divergence and intra-specific variation in three barcodes. Three barcodes of ITS2, ITS, *psb*A-*trn*H were analyzed for relative distribution of inter-specific divergence and intra-specific variation between *C. deserticola* and *C. tubulosa* based on the K2P genetic distance.

The barcoding gap presents the remarkable variation of inter- and intra-species, and demonstrates that separate, non-overlapping distributions between intra- and inter-specific samples. In this study (Fig. 5), the distance range was set to 0–0.45, because the greatest K2P distance of *psb*A-*trn*H between *C. deserticola* and *C. tubulosa* was close to 0.45. The three barcodes exhibited distinct gaps in the distributions of intra- and inter-specific variation. Furthermore, the gap of *psb*A-*trn*H was significantly larger than other two barcodes. Therefore, *psb*A-*trn*H region could be an ideal barcode for discriminating two origins of Cistanches Herba.

### Neighbor-joining (NJ) tree

**Figure 6:**
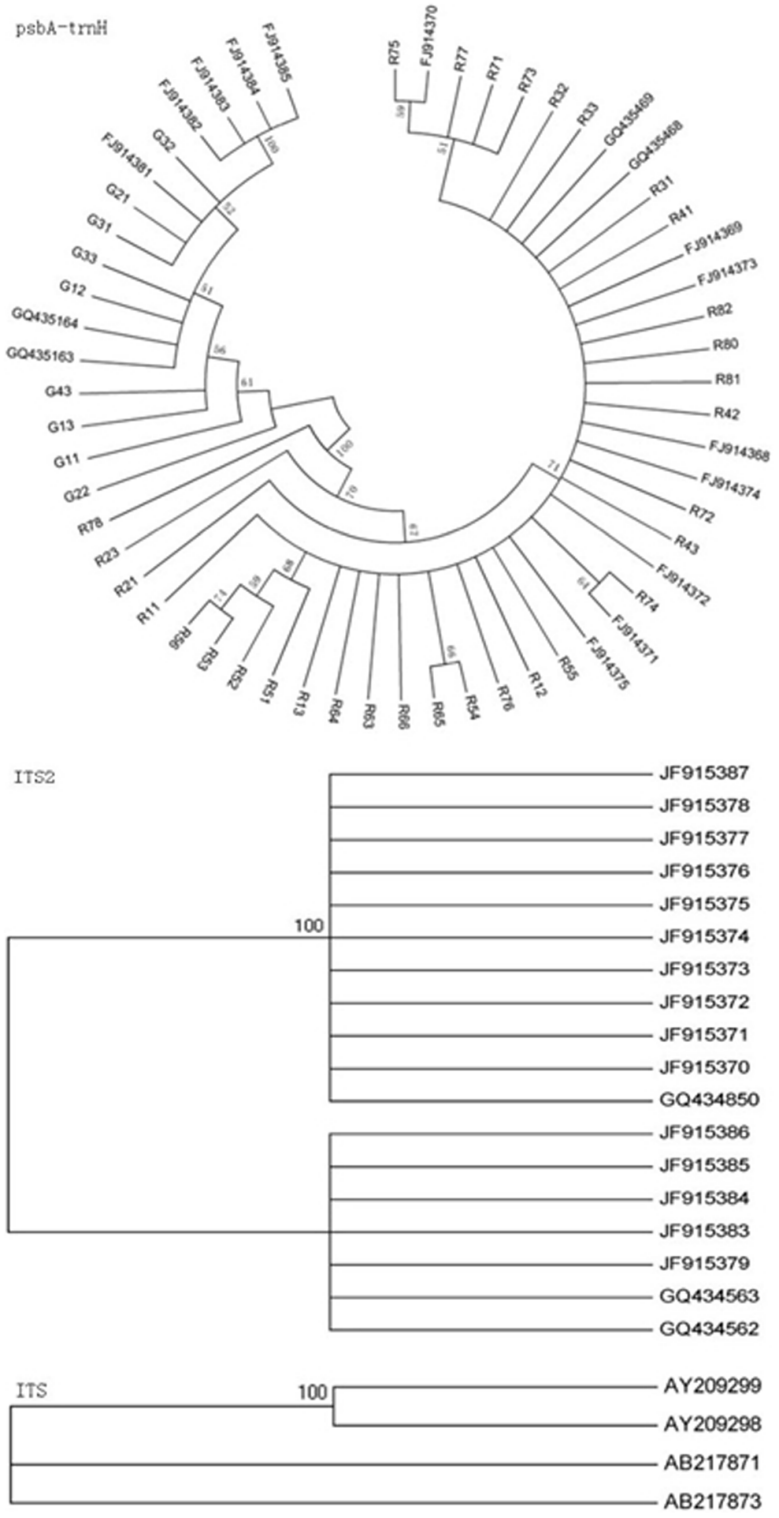
NJ tree of *C. deserticola* and *C. tubulosa* with three barcodes. NJ tree of *C. deserticola* and *C. tubulosa* with three barcodes were built. The bootstrap scores (1000 replicates) are shown (≥50%) for each branch. *C. deserticola* and *C. tubulosa* were clustered into two clades clearly.

An NJ tree illustrates the relationship among species and facilitates determination of their clustering. In this study, NJ tree of three barcodes were built based on K2P model (Fig. 6). The results demonstrated that two origins of Cistanches Herba clustered into two clades separately. Thus, the NJ tree clearly distinguished between *C. deserticola* and *C. tubulosa*.

## Discussion and Conclusions

Cistanches Herba is an important medicinal material commonly used to nourish in the Asian community [38]. However, the two origins of Cistanches Herba, *C. deserticola* and *C. tubulosa*, have different chemical compositions and pharmacological activities respectively. Concurrently, the two origins differ in clinical application and commodity market. The classification of *Cistanche* is confused and massive substitute and adulterants flood the market due to the shortage of resources and special growing environment for Cistanches Herba. Genus of *Cistanche* is accepted to include four species and one variant: *C. deserticola*, *C. tubulosa*, *C. sinensis*, *C. salsa*, and *C. salsa var. albiflora* [39]. Researchers in Japan considered the origin of Cistanches Herba as *C. salsa* [40]–[43], while it was identified as *C. deserticola* by Tu [44]–[46]. Therefore, it is confused in classification of *Cistanche*, and it is hard to discriminate the two origins of Cistanches Herba.

Traditional methods for quality control of Cistanches Herba are morphological identification [47], [48], microscopic identification [49] and TLC (Thin-Layer Chromatography) [50], [51], FTIR (Fourier Transform Infrared Spectroscopy) [14], HPLC (High Performance Liquid Chromatography) [52], [53]. Morphological and microscopic method can easily differentiate species from different genera or families that possess big difference in morphological and microscopic characteristics, while it is hard to distinguish sibling species. TLC and FTIR can clearly discriminate species that possess different kind of chemical compositions, whereas it is difficult to determine the chemical component and content. HPLC is mainly used for differentiating species with different chemical elemente contents, nevertheless, the time of analysis is longer and the sensitivity is relatively lower compared to UPLC. Correspondingly, UPLC-QTOF/MS technology was faster and more accurate in determining chemical composition than other chemical methods. Molecular identification methods exhibit well in discrimination based on the genetic variation, such as SDS-PAGE (Sodium Dodecyl Sulfate-Polyacrylamide Gel Electrophoresis) [54], AFLP (Amplified Fragment Length Polymorphism) [55], [56]. However, these molecular methods are not easy to operate and are not universal. Correspondingly, DNA barcoding could discriminate species more universally, quickly and accurately than other molecular methods. For the species from same genus and close genetic relationship, those methods alone may not perform well in identification. Here, we combined UPLC-QTOF/MS and DNA barcoding in identifying *C. deserticola* and *C. tubulosa*, and evaluated the chemical and molecular markers that would allow them to be discriminated. 23 qualified mass peaks were detected and 16 were identified by using UPLC–QTOF/MS, and three potential marker compounds were firstly found to facilitate the discrimination of two origins by PCA and OPLS-DA analysis. Furthermore,four indicators were assessed by DNA barcoding technology in terms of their ability to differentiate two origins: Identification efficiency, genetic efficiency, barcoding gap, and NJ tree analysis. The *psb*A-*trn*H region was supported as a suitable DNA barcode for discriminating *C. deserticola* and *C. tubulosa*.

In conclusion, we firstly established a new molecular and chemical analysis-combined method for discriminating and quality control in two origins of Cistanches Herba. DNA barcoding can discriminate two origins in genetic variation and authenticate species universally and accurately; UPLC-QTOF/MS technology can analyze chemical composition to evaluate the quality of medicinal materials rapidly and accurately. The combined method of DNA barcoding and UPLC-QTOF/MS technology guarantee the identification in multiple sources of medicinal materials more accurately and scientifically, and may serve as method for identifying other confusing species or genus in classification.
